# The Giant Mottled Eel, *Anguilla marmorata*, Uses Blue-Shifted Rod Photoreceptors during Upstream Migration

**DOI:** 10.1371/journal.pone.0103953

**Published:** 2014-08-07

**Authors:** Feng-Yu Wang, Wen-Chun Fu, I-Li Wang, Hong Young Yan, Tzi-Yuan Wang

**Affiliations:** 1 Taiwan Ocean Research Institute, National Applied Research Laboratories, Kaohsiung, Taiwan; 2 Sensory Physiology Laboratory, Marine Research Station, Academia Sinica, I-Lan County, Taiwan; 3 Chemical Biology and Molecular Biophysics, Taiwan International Graduate Program, Institute of Biological Chemistry, Academia Sinica, Taipei, Taiwan; 4 Institute of Biochemical Sciences, National Taiwan University, Taipei, Taiwan; 5 Hanse-Wissenschaftskolleg Institute for Advanced Study, Delmenhorst, Germany; 6 Biodiversity Research Center, Academia Sinica, Taipei, Taiwan; University of Florida, United States of America

## Abstract

Catadromous fishes migrate between ocean and freshwater during particular phases of their life cycle. The dramatic environmental changes shape their physiological features, e.g. visual sensitivity, olfactory ability, and salinity tolerance. *Anguilla marmorata*, a catadromous eel, migrates upstream on dark nights, following the lunar cycle. Such behavior may be correlated with ontogenetic changes in sensory systems. Therefore, this study was designed to identify changes in spectral sensitivity and opsin gene expression of *A. marmorata* during upstream migration. Microspectrophotometry analysis revealed that the tropical eel possesses a duplex retina with rod and cone photoreceptors. The λ_max_ of rod cells are 493, 489, and 489 nm in glass, yellow, and wild eels, while those of cone cells are 508, and 517 nm in yellow, and wild eels, respectively. Unlike European and American eels, Asian eels exhibited a blue-shifted pattern of rod photoreceptors during upstream migration. Quantitative gene expression analyses of four cloned opsin genes (Rh1f, Rh1d, Rh2, and SWS2) revealed that Rh1f expression is dominant at all three stages, while Rh1d is expressed only in older yellow eel. Furthermore, sequence comparison and protein modeling studies implied that a blue shift in Rh1d opsin may be induced by two known (N83, S292) and four putative (S124, V189, V286, I290) tuning sites adjacent to the retinal binding sites. Finally, expression of blue-shifted Rh1d opsin resulted in a spectral shift in rod photoreceptors. Our observations indicate that the giant mottled eel is color-blind, and its blue-shifted scotopic vision may influence its upstream migration behavior and habitat choice.

## Introduction

Fish habitats are highly diverse, ranging from the deep sea to the upper reaches of freshwater rivers in the mountains, and from the tropics to the Arctic; the photic conditions in these environments vary greatly in terms of turbidity, color, and brightness. Certain fishes can alter their visual abilities in different photic environments [1], [2]. For example, the spectral sensitivities of rod and cone photoreceptors of deep-water fishes adapt to match the blue-shifted spectral bandwidth of ambient light [3], [4]. In contrast, shallow-sea fishes, such as black bream, possess cone photoreceptors with higher maximal light absorbance wavelength (λ_max_) values to match their green light-dominated habitats [5].

Plasticity of sensory sensitivity is also crucial in speciation [6]–[9]. Aside from the variations between species or higher taxa, intraspecific differences in fish spectral sensitivity may arise from spatial adaptation or ontogenetic changes [4]. For example, in the sand goby, *Pomatoschistus minutus*, rod sensitivity is altered to fit the local light environment [10]. Furthermore, diadromous fishes, which migrate between freshwater and seawater, also exhibit adaptation to changes in their photic environment during development. As an example, Pacific salmon are born in freshwater rivers, mature in the ocean, and then return to their birthplace to spawn. In order to adapt to photo-environment changes from freshwater to seawater, the salmon express blue opsin insteand of UV gene(s) in the single cone [11]. Similarly, the spectral sensitivities of rod photoreceptors in two catadromous freshwater eels, European and American eels, are modified by alterations in chromophore and opsin gene usage during ontogenesis and spawning migration; it should be noted that spectral tuning via opsin shift (rather than A1/A2 shifts) was first observed in eels [12]–[16].

The visual system plays important roles in foraging, prey capture, predator avoidance, and mating behavior. Two types of photoreceptors are found in fish retinas - rod and cone cells. Rod cells mediate scotopic vision, while cone cells mediate photopic, high acuity vision [17]. Several molecular mechanisms have been demonstrated to alter the spectral sensitivity of photoreceptors in fish. First, the spectral sensitivity of visual pigments can be modulated by differential expression of five classes of opsin genes, including rhodopsin (Rh1) in rod cells and four other genes in cone cells [18]–[20]. Second, the λ_max_ of the visual pigment changes depending on whether it uses 11-cis-retinal (vitamin A1-derived) or 3-dehydroretinal (vitamin A2-derived) chromophores [1], [21]–[23]. Third, amino acid substitutions within opsins can result in the spectral shift of visual pigments [24]–[26]. Based on crystal structure analysis and mutagenesis studies, it is known that amino acid changes at 26 sites are involved in the spectral tuning of visual pigments in vertebrates [2], [27]. Finally, several studies have shown that accumulation of interactions between distally-located amino acid substitutions and the retinal binding pocket may also induce spectral shifts [28], [29]. Therefore, a modeling study based on available opsin gene sequences may yield useful information with regard to the interplay of tuning sites and spectral shifts.

Freshwater eels are born in the deep-sea, mature in freshwater, and then return to the deep-sea to spawn. However, many studies have shown that some populations of temperate eels never enter freshwater, but stay in estuarine and coastal waters until maturation [30]–[36]. Similar plasticity in migratory behavior was also observed in tropic freshwater eels, i.e. giant mottled eel (*Anguilla marmorata*) and bicolor eel (*A. bicolor*) [37]. This plasticity may be influenced by intra- or inter-specific competition [34]. For example, the giant mottled eel and Japanese eel are considered sympatric in Taiwan. However, otolith microchemistry studies have shown that the giant mottled eel is more abundant in the upper reaches of the rivers, while the Japanese eel preferentially inhabits lower reaches or estuaries within the same river [34], [38]. This disparity in migratory behaviors and habitat choice between species may reflect inter-specific competition or selection for certain environmental parameters.

To date, our understanding of the ontogenetic changes of spectral sensitivities of freshwater eels are based on studies of temperature eels, including European and American eels. These species exhibit a spectral shift towards red during upstream ontogenetic migration, but a shift towards blue during downstream spawning migration [12], [15]. In contrast, such studies using tropic eels are limited. The current study was devised to test the hypothesis that migration behavior or habitat choices affect the spectral sensitivity and opsin gene expression of giant mottled eels during their upstream ontogenetic migration. In addition, the interactive forces of amino acids within cloned opsins were predicted and analyzed, to investigate the mechanisms of spectral tuning. Finally, opsin gene expression patterns and photoreceptor spectral sensitivities at different developmental stages of the eel were determined. Our findings thus reveal the mechanisms of the ontogenetic changes in the visual system of giant mottled eel.

## Materials and Methods

### Sampling localities and collections

To study spectral sensitivity, clone opsin genes, and quantify gene expression, giant mottled eels of different developmental stages were collected. The glass-stage eels (denoted as ‘Glass’) were collected from the mouth of the Hsiukuluan River (23°27′41.9′′N 121°30′05.2′′E) in eastern Taiwan. Yellow eels were collected in two different ways: four specimens were bought from an eel farm (22°44′50.1′′N 120°32′43.0′′E) and consent/permission was obtained from an eel farm owner in Pingtung County (the eels were 3-years-old, and are denoted as ‘Cultured yellow’), while two specimens were caught upstream of the Laomei Stream (25°15′25.9′′N 121°32′11.4′′E) in northern Taiwan (these eels are denoted as ‘Wild yellow’). During the two field collections (Hsiukuluan River and Laomei Stream), an Ocean Optics UBS-2000 spectrophotometer with a waterproof probe was used to measure the *in situ* light spectra 30-cm underwater in order to provide photic parameters of the environments where the samples resided (Figure S4). The sample sizes for each stage were as follows: 9 for Glass, 4 for Cultured yellow, and 2 for Wild yellow (Table S1). Specific permission was not required to obtain the indicated animals from these field locations for the activities described. The field studies did not involve endangered or protected species. For studies of spectral sensitivity, specimens were kept alive in a tank with running freshwater (temperature of 25∼28°C) under a natural light cycle. Animal use protocols No. RFiZOOYH2007012 & IACUC_11-02-133, approved by the Academia Sinica Institutional Animal Care and Use Committee (IACUC), were followed for all surgical procedures to minimize suffering.

### Microspectrophotometry (MSP)

After overnight dark adaptation inside a darkroom, eels were first anesthetized with an overdose of MS-222 (50 ppm), and then enucleated under a dim red light. Retinae were removed under a stereomicroscope by technicians wearing night vision goggles, and were immediately immersed in chilled phosphate buffered saline with 6% sucrose, (Sigma, USA; pH 6.5). Retinae were cut into pieces, placed between two cover slides (20 mm×30 mm), sealed with silicone grease, and placed onto the single-beam, computer-controlled, microspectrophotometer stage to measure the absorbance spectra of photoreceptors [39], [40]. The absorbance curve and the wavelength of maximal absorbance (λ_max_) of photoreceptors were obtained by a programmed statistical method [40]. Examples of absorbance curves are presented in Figure S1. The λ_max_ and A1/A2 template of the normalized absorbance spectra were determined followed a previously described method [41]–[43]. For each measurement, the best template of fit was obtained using a visual examining procedure. The best visual fit was the template with the lowest standard deviation (SD). If the SD of the λ_max_ was smaller than 7.5 nm, then the spectrum was considered valid and collected for analysis [44], [45]. Approximately 40 measurements were obtained from each specimen. The λ_max_ values of each photoreceptor were averaged, and then a final estimate of mean λ_max_ ± SD of each category of retinal cell was obtained.

### Extraction of genomic DNA and total RNA, and cDNA synthesis

Genomic DNA was extracted from 100 mg of muscle tissue using a Roche DNA Isolation Kit (Indianapolis, USA), following the manufacturer's instructions. The heads of glass-stage eels and the eyecups (without the lens) of yellow eels were collected and immersed in RNAlater (Ambion, Inc., Austin, TX) and stored at −80°C. Total RNA was extracted using the Qiagen RNeasy mini kit (Valencia, USA). To prevent contamination by genomic DNA, RNA was treated with TURBO DNase (Ambion, Inc., Austin, TX). Total RNA was reverse-transcribed to cDNA using the SuperScript III First-Strand Synthesis System (Invitrogen, Carlsbad, California, USA) with oligo-d(T) primers. The cDNA was used as template for PCR.

### Opsin gene cloning and sequencing

Target genes were amplified with the indicated primers (see below) and genomic DNA as template using a Fast-Run Taq Master Mix (Protech Technology Enterprise Co., Taiwan), in accordance with the manufacturer's recommendations. The PCR products were cloned individually into the pGEM-T vector (Promega, Madison, USA), and five to ten clones were sequenced to ensure fidelity. All primers and the accession number of cloned genes are listed in Table S2 and Table S3, respectively. The cloning protocols for each type of gene are described in detail below:

House-keeping genes: mitochondrial *cytochrome b* (intron-free) and acidic ribosomal phosphoprotein P0 (ARP) were selected as house-keeping genes to serve as endogenous controls for normalization of quantitative PCR data (Weltzien *et al.* 2005). The primers used to amplify these house-keeping genes in *A. marmorata* were designed based on those of *A. anguilla* [46].

Rod opsin genes: retinal cDNA and genomic DNA were used as templates to amplify Rh1f (freshwater type) and Rh1d (deep-sea type) opsin genes, respectively. The P1-P2/P1-P3 primer sets originally designed to amplify the fwo and dso opsin genes of *A. japonica* [47], were used to amplify rod opsin genes.

Cone opsin genes: complete cone opsin mRNA sequences were obtained as described by Carleton & Kocher (2001). A degenerate primer set, OPF and OPR, was used to clone an exon region of the cone opsin genes; the *A. marmorata* opsin gene sequences were used as a basis for designing the gene-specific primers to amplify the 5′- and 3′-RACE fragments. Opsin genes were cloned from retinal RNA using 3′ and 5′ rapid amplification of cDNA ends (RACE) with the SMART^™^ RACE Amplification Kit (Clontech Laboratories, Inc., USA), following the manufacturer's instructions. The resulting products were gel-purified, cloned, sequenced, and assembled.

### Phylogenetic analysis of ospin genes

The sequences of *cytochrome b* and opsin genes were downloaded from Genbank for comparison and analysis (Table S3). The Clustal W function merged in MEGA 5 software [48] was used to align the sequences according to the predicted amino acid sequences. The best-fit model of nucleotide substitution was determined by hierarchical likelihood ratio tests (LRT) implemented in Model Test v3.7 [49]. Neighbor-joining [50] trees of each gene were constructed using PAUP 4.0* [51] and MEGA 5 software with the best-fit model of nucleotide substitution and 1000 bootstrap replicates. Ancestral sequences of the opsin genes of freshwater eels were predicted using PAML 1.4 [52].

### Rhodopsin structure prediction

To investigate possible tuning sites, we applied SwissModel and Ligplot to predict the protein structure of Rh1 and the amino acid interactions/interactive forces. Protein models of eel rhodopsins (Rh1) were constructed with SwissModel (http://swissmodel.expasy.org/) with the X-ray structure of bovine rhodopsin (PDB code: 1U19) as template. The 3D structure simulation could reveal the functional amino acids for putative tuning sites. In addition, the hydrogen bonds and hydrophobic interactions between amino acid residues and the retinal were analyzed with Ligplot software (http://www.ebi.ac.uk/thornton-srv/software/LIGPLOT/). The Ligplot diagrams portray the hydrogen-bond interaction patterns and hydrophobic contacts between the ligand(s) and the main-chain or side-chain elements of the protein. The Ligplot system is able to analyze a single ligand binding to homologous proteins, or general cases in which both the protein and ligand change. These analyses revealed putative tuning sites of Rh1 opsins.

### Quantitative PCR

Relative gene expression ratios were analyzed by quantitative PCR. Gene specific primer pairs were designed using the Primer Express software from Applied Biosystems (Carlsbad, California, USA). The amplification efficiency of the opsin genes and the house keeping genes were approximately equal. Quantitative PCR analyses were carried out in a final volume of 20 µl, which contained 3 µl diluted cDNA (10 ng/µl), 0.5 µl each of gene-specific forward and reverse primers (5 µM), and 10 µl Fast SYBR Green Master Mix from Applied Biosystems (Carlsbad, California, USA); thermal cycling was performed as follows: 40 cycles of denaturation at 95°C for 3 sec and annealing/extension at 60°C for 30 sec. The relative expression of each opsin gene was calculated with the following equation: 
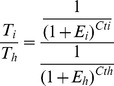
where T_i_ is the expression level for a given gene I; T_h_ is the expression level for the relevant house-keeping gene; E_i_ is the PCR efficiency for each opsin gene primer set; E_h_ is the PCR efficiency for the house-keeping gene primer set; and Cti and Cth are the critical cycle number for each opsin gene and house-keeping gene, respectively.

Proportional opsin expression was calculated as a fraction of total opsin gene expression for an individual, according to the following equation:
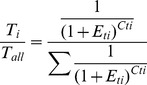
where T_i_ is the proportional expression for a given gene i; T_all_ is the total expression for a given gene i; E_ti_ is the PCR efficiency for each primer set; and Cti is the critical cycle number for each gene. The QPCR primer sequences are shown in Table S2.

## Results

### Spectral sensitivities of photoreceptor cells

The mean λ_max_ values of rod cells were 493±4.7 nm for glass eels and 489±6.1 nm for cultured yellow eels (Table 1). No significant difference in λ_max_ was observed between cultured and wild yellow eels (489±5.1nm). The λ_max_ of glass eel was significantly different from those of other stages (Table 2). A spectral shift of 4 nm was observed between glass eels and eels at other stages.

**Table 1:** The mean λ_max_ of photoreceptor cells from *A. marmorata* at different developmental stages, as measured using MSP.

Developmental stage	Rod cells	Cone cells (Green single cone)
**Glass eel (N = 3)**	493±4.7 nm (n = 60)	Not detectable
**Cultured yellow eel (N = 4)**	489±6.1 nm (n = 40)	508±8.0 nm (n = 9)
**Wild yellow eel (N = 2)**	489±5.0 nm (n = 44)	517±14.2 nm (n = 7)

All values are expressed in nanometers (nm) ± SD. The number of photoreceptor cells measured is indicated in parentheses. N and n indicate the number of specimens and cells examined, respectively.

**Table 2:** Comparisons of the spectral sensitivities of rod and cone cells at different developmental stages of *A. marmorata* by *t*-test.

	Glass eels	Cultured yellow eels	Wild yellow eels
**Glass eels**		*	*
**Cultured yellow eels**			NS
**Wild yellow eels**		NS	

The upper-right half presents the *t*-test results for rod cells, while the lower left half presents those for cone cells.* indicates P<0.05; NS, no significant difference: P>0.05.

**Figure 1:**
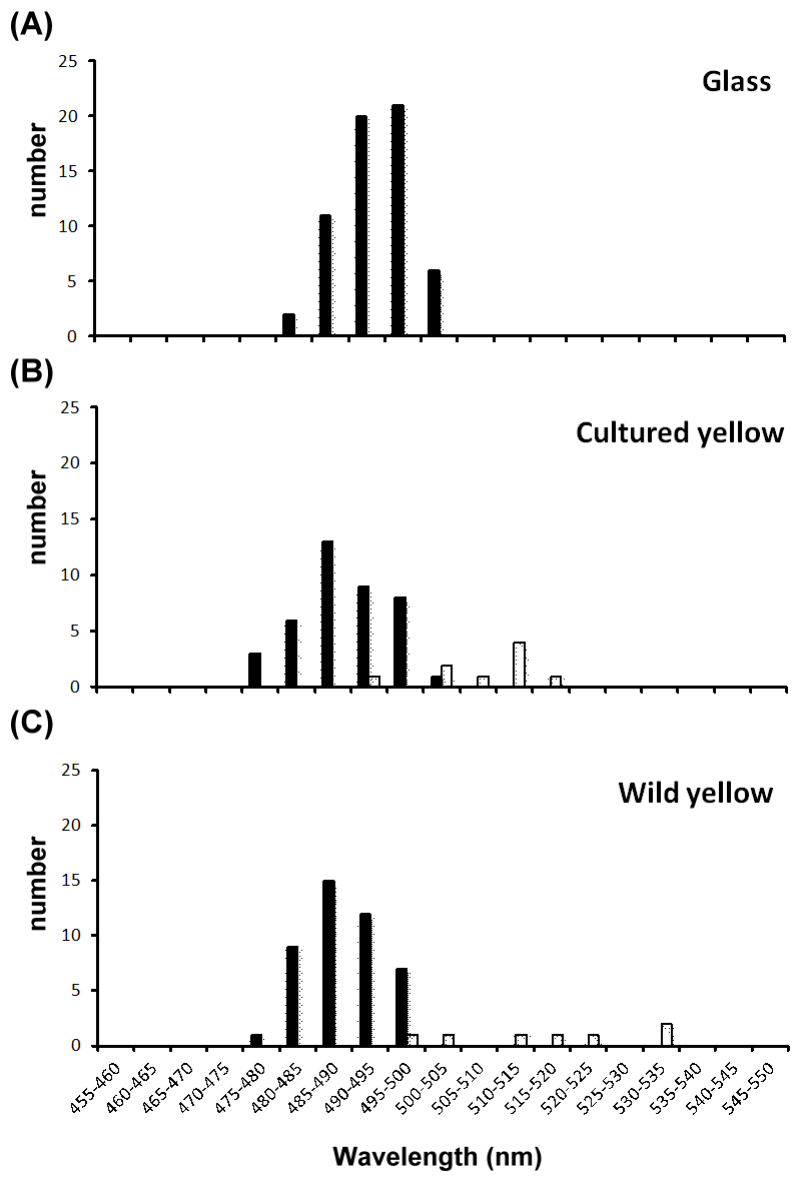
Distribution histograms of the λ_max_ of photoreceptor cells at four stages of *A. marmorata*. (A) Glass eel; (B) Cultured yellow eel; (C) Wild yellow eel. Rod cells: black bars. Cone cells: white bars.

Only a single cone cell was observed in cultured and wild yellow eels. The mean λ_max_ values of single cone cells are all in the range of green light spectra (Table 1). Student's *t*-test showed that the λ_max_ values between cultured yellow eel and other stages are significantly different (Table 2). The λ_max_ frequency distributions for rod and cone cells at each stage are presented in Figure 1.

### Classification of freshwater eel opsins and phylogenetic analysis

Four opsin genes were cloned from retinal cDNA and/or genomic DNA. Two types of rod opsin genes, Rh1f (freshwater type) and Rh1d (deep-sea type), were obtained, both of which were 1025 base pairs (bps) in length. The Rh1f gene is usually expressed in eels during the juvenile stage in freshwater, while the Rh1d gene is expressed during spawning migration to the deep-sea [47], [53]. We also identified two types of cone opsin genes, SWS2 (short-wavelength sensitive 2, blue-sensitive) and Rh2 (rhodopsin-like, green-sensitive), which encode mRNAs of 2459 and 2306 bps in length, respectively; the coding regions were 1080 and 1044 bps, respectively.

**Figure 2:**
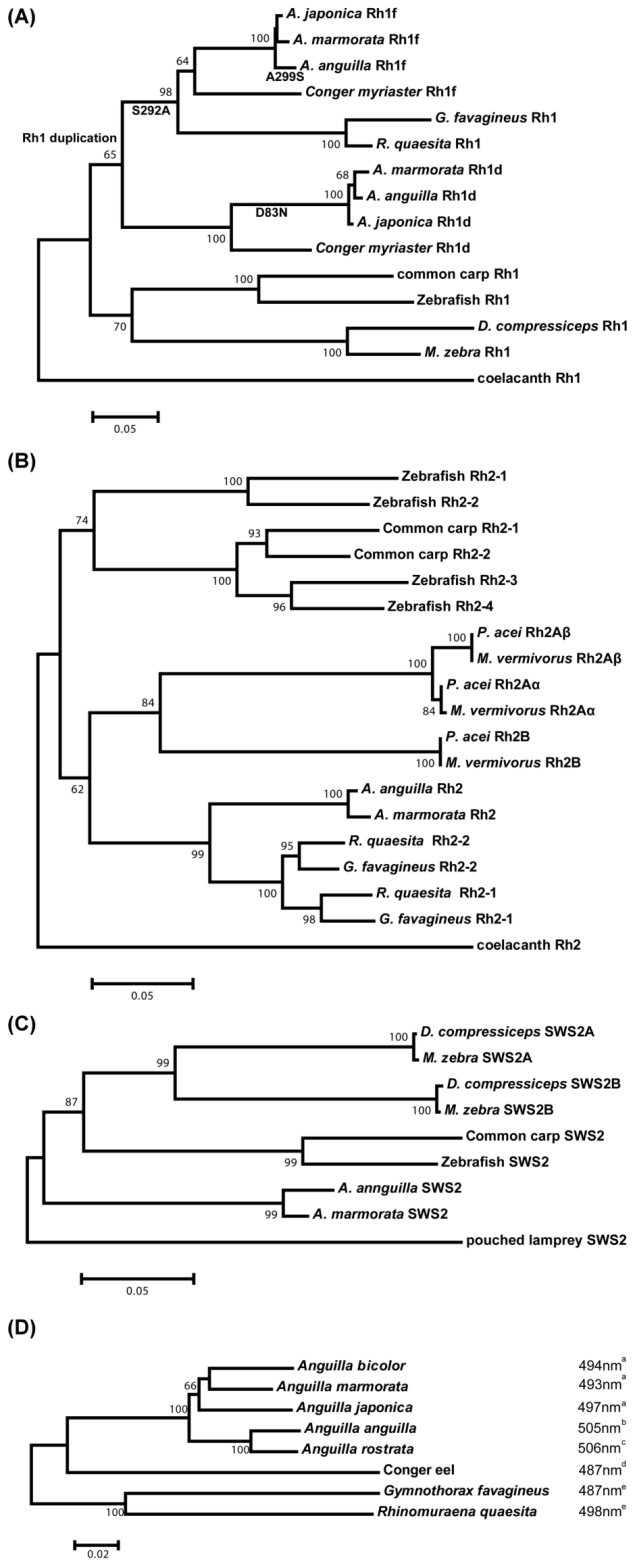
Neighbor joining trees of the freshwater eel Rh1 (A), Rh2 (B), and SWS2 (C) opsin and (D) *cytochrome b* genes based on Maxima-likelihood distances. The scale bar represents 0.05 nucleotide substitutions. The nucleotide sequences of fish opsin genes were obtained from GenBank. The genes and their accession numbers are listed in Table S3. ^a^ λ_max_ of rod cells from Table 1 & Table 6. ^b^ λ_max_ of rod cells from [15]. ^c^ λ_max_ of rod cells from [12]. ^d^ λ_max_ of rod cells from [71]. ^e^ λ_max_ of rod cells from [65].

Few opsin gene sequences have been characterized in Anguilliform fishes; as such, we used opsin genes from cichlids, cyprinids, salmons, lamprey, and coelacanth as substitutive out-groups for phylogenetic analysis (Figure 2). The Rh1 genes of freshwater eels were separated into Rh1f and Rh1d clades (Figure 2A). *A. marmorata* is clustered with *A. anguilla* in the Rh1d clade, but is clustered with *A. japonica* in the Rh1f clade. The results suggest two possible different evolutionary origins for the Rh1 genes of freshwater eels. For Rh2, the freshwater eel genes are the sister group of moray eel genes, and these form a distinct monophyletic group from other fishes (Figure 2B). For SWS2, freshwater eel genes are clustered together, and located at the basal position of the phylogenetic tree (Figure 2C). For *cytochrome b*, the freshwater eel genes form a monophyletic group (Figure 2D).

### Comparison of putative spectral tuning sites

We subsequently compared the cloned opsin gene sequences with those of two freshwater eels (*A. anguilla* and *A. japonica*), as shown in Table 3 and 4. In the Rh1 gene, there are seven amino acid sites important for spectral tuning: positions 83, 122, 207, 211, 265, 292, and 295 [54]. Tuning sites in the Rh1d and Rh1f genes of freshwater eels are identical, with the exceptions of positions 83 and 292. Earlier studies utilizing site-directed mutagenesis suggested that the S292A and A292S substitutions in vertebrates may induce a 7–16 nm red-shift and a 7–15 nm blue-shift of λ_max_, respectively [27], [53], [55]–[57]. Moreover, the D83N substitution may cause a blue-shift in the λ_max_ of Rh1 in fishes [27], [58]. In Rh2, four specific amino acid sites have been reported to be involved in spectral tuning: positions 97, 122, 207, and 292 [25], [27]; these sites are fully conserved among the freshwater eels. In SWS2, substitutions at amino acid sites 94, 116, 118, 265, and 292 can result in spectral shifts. Here, we only observed one substitution (M116T) between the SWS2 genes of *A. marmorata* and *A. anguilla*; however, it should be noted that only one SWS2 gene from *A. anguilla* was available for comparison.

**Table 3:** Comparisons of the Rh1 sequences of freshwater eels.

Gene	Rh1						
	Amino acid Sites						
	83	122	207	211	265	292	295
Consensus	D	E	M	H	W	S	A
*A. anguilla Rh1f*	.	.	.	.	.	A	.
*A. japonica Rh1f*	.	.	.	.	.	A	.
*A. marmorata Rh1f* *	.	.	.	.	.	A	.
*A. anguilla Rh1d*	N	.	.	.	.	.	.
*A. japonica Rh1d*	N	.	.	.	.	.	.
*A. marmorata Rh1d* *	N	.	.	.	.	.	.

* indicates the opsin gene obtained in this study.

Sequences are compared to the consensus sequence, with identical amino acids indicated by a dot. Sites are numbered according to bovine rhodopsin.

**Table 4:** Comparisons of the Rh2 and SWS2 sequences of freshwater eels.

Gene	Rh2				Gene	SWS2				
	Amino acid sites					Amino acid sites				
	97	122	207	292		94	116	118	265	292
Consensus	T	E	M	S	Consensus	A	M	T	W	S
*A. anguilla*	.	.	.	.	*A. anguilla*	.	.	.	.	.
*A. marmorata* *	.	.	.	.	*A. marmorata* *	.	T	.	.	.

* indicates the opsin gene obtained in this study.

Sequences are compared to the consensus sequence, with identical amino acids indicated by a dot. Sites are numbered according to bovine rhodopsin.

### Opsin gene expression at different life stages

**Figure 3:**
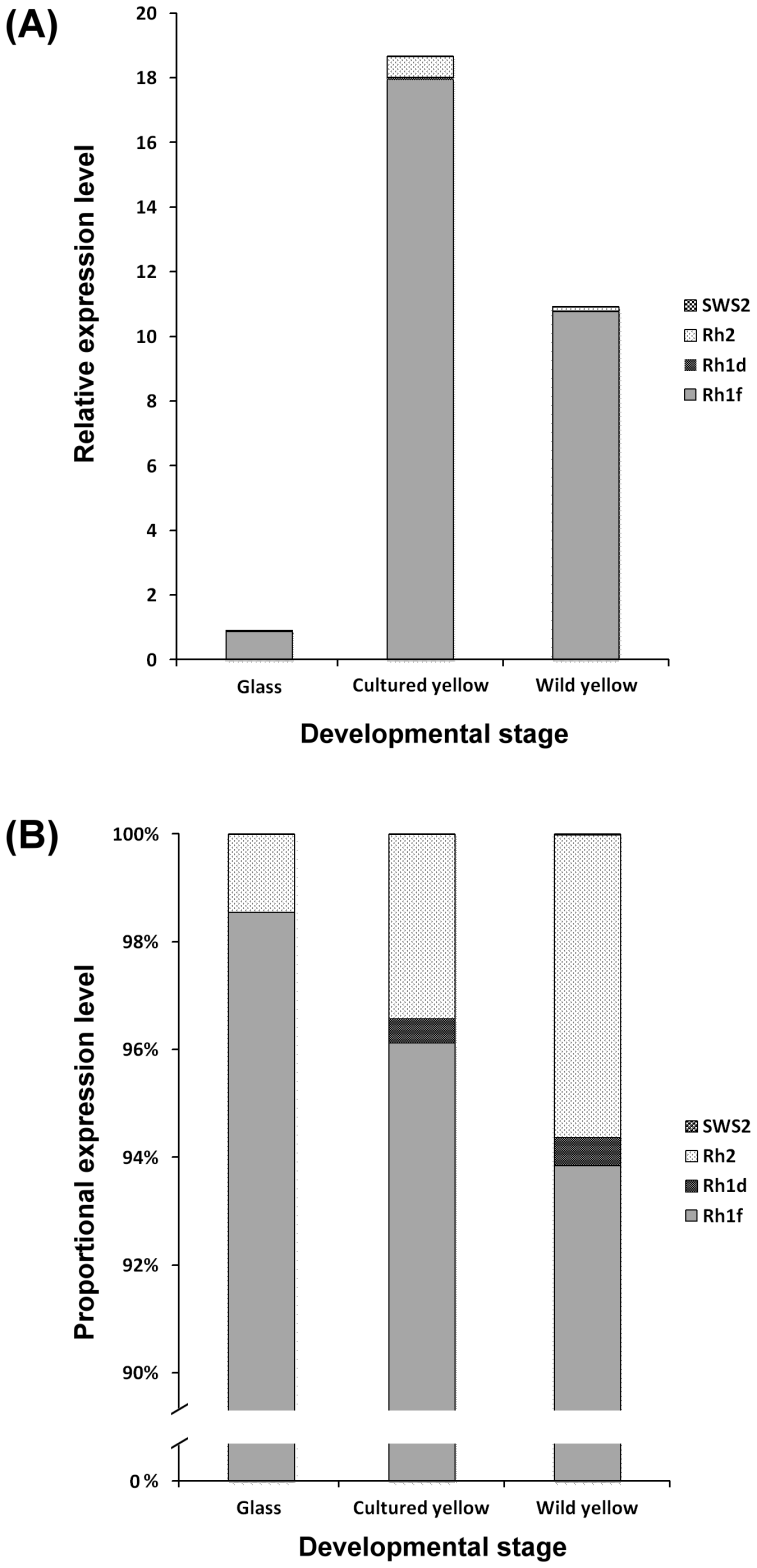
The relative expression (A) and proportional expression (B) of opsin genes at different developmental stages of *A. marmorata*, as determined by quantitative RT-PCR of retinal RNA.

The relative expression levels of total opsin genes in more mature stages (cultured and wild yellow eels) are significantly higher than those in glass-eel stage. Rh1f was found to be the dominant opsin gene expressed at all stages (Figure 3A). Rh1f expression accounted for 93.6 to 98.5% of total opsin gene expression, while Rh2 gene expression accounted for 1.5∼6.3% (Figure 3B). Rh1d was only expressed in older eels (cultured and wild yellow eels), while SWS2 was not detected at any stage. These findings suggest that *A. marmorata* may require Rh1f and Rh2 opsin during upstream migration, and this observation is consistent with the MSP data.

### Rh1 protein structure analysis reveals putative tuning sites

We proceeded to predict and compare the amino acid interactions/interactive forces within the Rh1f pigments of freshwater eels (Table 5). The results of prediction and modeling confirmed that two known tuning sites, D83N and A292S, interact with different residues depending on the protein, and may thus influence Rh1 protein structure (Figure S2). The same criteria predicted that four amino acid residues (A124S, I189V, I286V, and L290I) near the retinal binding pocket may be putative tuning sites, and that the interactions of these sites also differ between Rh1f and Rh1d (Figure S3). In addition, we observed that eight amino acid residues (sites 112, 137, 189, 191, 193, 219, 255, and 313) interacted with different amino acid residues depending on the protein, and interacted indirectly with residues near the retinal molecules.

**Table 5:** Eight putative tuning sites of Rh1f, as predicted using protein modeling and Ligplot.

Central site	Species	Amino acid	Hydrogen bond	Hydrophobic interaction
**112**	*A. marmorata* b	L	Thr 108, Phe 116	-
	*A. japonica*	I	Thr 108, Phe 116	Gly 89, Gly 90
	*A. Anguilla*	I	Thr 108, Phe 116	Gly 89, Gly 90
**137**	*A. marmorata*	V	Ile 133	Lys 141, Pro 142, Phe 146, Phe 148
	*A. japonica*	V	Ile 133	Lys 141, Pro 142, Phe 146, Phe 148
	*A. Anguilla*	M	Val 133	Lys 141, Pro 142, Phe 146, Phe 148
**189** c	*A. marmorata*	I	-	Pro 171, Try 178, Tyr 191, Phe 203, Ret 1296
	*A. japonica*	I	-	Pro 171, Try 178, Tyr 191, Phe 203, Ret 1296
	*A. anguilla* b	M	-	Thr 118, Cys 167, Phe 203, Met 207, Ret 1296
**191**	*A. marmorata*	Y	Tyr 268	Ile 189, Ala 272
	*A. japonica*	Y	Tyr 268	Ile 189, Ala 272
	*A. anguilla* b	H	-	Val 204, Met 297, Phe 208
**193**	*A. marmorata*	T	Arg 177, Asp 190	-
	*A. japonica*	T	Arg 177, Asp 190	-
	*A. anguilla* b	A	Asp 190	-
**219**	*A. marmorata*	V	Pro 215, Tyr 223	Val 129
	*A. japonica*	V	Pro 215, Tyr 223	Val 129
	*A. anguilla* b	I	Pro 215, Tyr 223	Leu 128, Val 129
**255**	*A. marmorata*	V	Thr 251, Val 258, Ile 259	-
	*A. japonica*	V	Thr 251, Val 258, Ile 259	-
	*A. anguilla* b	I	Thr 251, Val 258, Ile 259	Tyr 223
**313**	*A. marmorata*	F	Asn 310, Met 317	Val 61, **Tyr 306**, Ile 307
	*A. japonica*	F	Asn 310, Met 317	Val 61, **Tyr 306**, Ile 307
	*A. anguilla* b	S	Asn 310, Met 317	-
**retinal** a	*A. marmorata*	Retinal	-	Ala 117, Gly 121, Gly 188, Ile 189, Met 207, Phe 212, Phe 261, Trp 265, Tyr 268, Ala 269
	*A. japonica*	Retinal	-	Ala 117, Gly 121, Gly 188, Ile 189, Met 207, Phe 212, Phe 261, Trp 265, Tyr 268, Ala 269
	*A. Anguilla*	Retinal	-	Ala 117, Gly 121, Gly 188, Met 189, Met 207, Phe 212, Phe 261, Trp 265, Tyr 268, Ala 269

a the retinal molecule is covalently bound to Lys 296.

b the number of binding amino acids, as predicted by Ligplot.

c site 189 may directly bind to the retinal molecule, as predicted by Ligplot.

## Discussion

### Color-blindness and blue-shifted visual spectra during upstream migration

To distinguish between colors, an organism must possess at least two cone photoreceptors with distinct spectra. Species with only one type of color receptor in their retina are regarded as having “monochromatic vision” or being color-blind [1], [59]. Here, we report that *A. marmorata* possesses only one type of cone cell, which senses a limited range of the light spectrum, i.e., from 500 nm to 535 nm (Figure 1). Furthermore, our quantitative PCR data demonstrate that Rh2 is the only green cone opsin gene expressed at all stages (Figure 3B). These findings clearly indicate that the giant mottled eel is color-blind during upstream migration. However, European eel (*A. anguilla*) possesses green and blue cones at the large yellow eel stage (older than 4 years old), suggesting it may be able to discriminate colors [60]. Cottrill *et al.* (2009) further showed that blue cone opsin genes are expressed at low levels during the silver eel stage of European eel. The varying abilities of freshwater eels to discriminate color may result from the plasticity of ontogenetic expression and/or migratory behavior.

The MSP results demonstrate that the rod cells of *A. marmorata* exhibit a blue-shifted pattern during upstream migration (Figure 1). The λ_max_ value of rod cells decreased from 493 nm in glass eels to 489 nm in yellow eels (Table 1). This decrease may result from adaptation to different photic environments. Water at the river mouth area is usually turbid and dominated by longer wavelength light, i.e., red light [61]; however, water upstream is usually clear and dominated by light with shorter wavelengths, such as blue light. To optimize vision, rod cell spectral sensitivity may be shifted during upstream migration of *A. marmorata*. The observed 4 nm rod cell spectral shift may be a consequence of the expression of alternative opsin genes. We found that Rh1f gene expression dominated at all sampled stages, but Rh1d gene expression was detectable only in yellow eels; these findings may account for the 4 nm spectral shift of rod cells during development.

### Plastic migratory behaviors and spectral sensitivity in freshwater eels

The spectral sensitivity of *A. marmorata* alters as it matures. At the glass eel stage, the average λ_max_ of *A. marmorata* rod cells was 493 nm, whereas that of European eel rod cells was 505 nm [15]. By the yellow eel stage, the average λ_max_ of *A. marmorata* rod cells shifted to 489 nm, while American and European eels of the same stage possessed rod cells with λ_max_ values of around 516 nm [12], [13]. Therefore, significant red shifts occur in the rod cells of European and American eels during upstream migration, as opposed to the blue shift of *A. marmorata* rod cells.

Several studies have shown that freshwater eels have a flexible migratory life cycle (see the Introduction). Temperate freshwater eels, (i.e., European, American, and Japanese eels) have been described as exhibiting one of the following migratory strategies: (1) restricted to freshwater; (2) restricted to brackish water; (3) frequent migration between estuaries and the ocean. Tropic freshwater eels (i.e., giant mottled eel and bicolor eel) exhibit plastic strategies of upstream migration. Furthermore, *A. marmorata* may preferentially inhabit environments containing either multiple or single species of *Anguilla* sp. [30]. In Taiwan, *A. marmorata* and *A. japonica* are sympatric. Based on Sr/Ca ratio analysis of otoliths, *A. marmorata* prefers to live in the upper reaches of rivers, while *A. japonica* favors the lower reaches or estuaries within the same river [34], [38]. Such differences in migratory behaviors and habitat choice between species may reflect their ability to adapt to varying visual signals.

While photic conditions at the river mouth are usually turbid, those upstream are usually clear [61]. Several studies have shown that fishes which inhabit turbid water tend to possess photoreceptors with longer λ_max_ values than those of fishes that live in clear water [21], [43], [62]. In turbid water, short-wavelength light is readily absorbed by particulates, and therefore cyprinids, which live in such habitats, have evolved photoreceptors that detect longer wavelengths. In this study, the wild yellow eels were collected from a small stream in the upper basin of the Laomei stream. The water of this sampling location is clearer than that of estuaries, where the glass eels were collected. The Figure S4 shows that the water of the upper basin of Laomei stream exhibits a bluer spectrum than the water of the estuary of the Hsiukuluan River. Such a phenomenon indicates that the upper basin of the river presented a bluer photic environment. In addition, we observed that the λ_max_ value of *A. japonica* rod cells is 497.5 nm, slightly longer than those of *A. marmorata* and *A. bicolor* (Table 6). The former two eels have similar spawning areas and migratory behaviors, but inhabit different environments [34], [36], [37]. Therefore, we suggest that blue-shifted visual spectra may facilitate adaptation of giant mottled eel to clear water during upstream migration.

**Table 6:** The mean λ_max_ of rod cells from *A. japonica* and *A. bicolor pacific* glass eels, as determined using MSP.

Species	Rod cells	Cone cells (Green single cone)
*A. japonica* (N = 2)	497±5.3 nm (n = 32)	510±7.4 nm (n = 3)
*A. bicolor pacific* (N = 4)	494±5.2 nm (n = 37)	514±9.8 nm (n = 18)

All values are expressed in nanometers (nm) ± SD. The number of photoreceptor cells measured is indicated in parentheses.

N and n indicate the number of specimens and cells examined, respectively.

### Spectral shift of rod cells between European and giant mottled eel

Comparing the MSP results of this study to those of a previous study of European eel revealed a 10 nm spectral shift between the rod cells of *A. marmorata* and *A. anguilla* at the glass eel stage. The possible mechanisms were introduced previously. We will address four possibilities in turn. First, examination of opsin gene expression in *A. marmorata* (this study) and *A. anguilla* [46] revealed that Rh1f is the most highly expressed opsin gene, while Rh1d is undetectable at the glass eel stages. Second, we describe here that freshwater eels share the same substitutions at known spectral tuning sites of Rh1f opsin (Table 3). Third, both template fitting in this study (Table S4) and the HPLC analysis of a previous study [15] revealed that A1-retinal predominates at the glass eel stages of *A. marmorata* and *A. anguilla*. However, the A1/A2 ratio in *A. marmorata* is 3∶1, while that in *A. anguilla* is 6∶4. The giant mottled eel contained more A1-retinal than European eel at all stages. Therefore, alternative chromophore usage may induce the spectral shift of rod cells. Finally, fourteen amino acid substitutions were observed between the Rh1f opsins of *A. marmorata* and *A. anguilla*, but none of these are located at known tuning sites. Earlier studies have shown that the accumulation of distal effects can induce spectral shifts of opsin pigments in vertebrates [28], [29]. Our structural model predicts that the amino acid interaction/interactive forces of eight sites differ between the Rh1f opsins of *A. anguilla* and *A. marmorata* (Table 5). Four of them, M137V, M189I, H191Y and I255V, are involved in adjusting the spectral sensitivity of opsin pigments [63], [64]. As mentioned above, alternative chromophore usage and amino acid substitutions located distally to the retinal binding pocket may cause spectral shifts of Rh1f opsin between *A. marmorata* and *A. anguilla*.

### Evolution of opsin genes in freshwater eels

The phylogenetic tree of the Rh1 gene (Figure 2A) reveals that a duplication event occurred before the speciation of freshwater, moray, and conger eels. The predicted ancestral sequence of the Anguilliformes Rh1 gene (Figure S5) contains serine at site 292; furthermore, the λ_max_ of Rh1 in these species is usually around 485 nm [3]. The S292A substitution, which induced a red-shift in Rh1 opsin, occurred in the freshwater eel Rh1f lineage. On the other hand, the D83N substitution, which induced a blue-shift in Rh1 opsin, occurred in the freshwater eel Rh1d lineage. In addition, our analyses have demonstrated that eight putative tuning sites can affect Rh1f opsin function. These results imply that the ancestors of freshwater eels possessed rod cells capable of adapting to different photic environments during catadromous migration.

Rh2 gene duplication has been regarded as a common occurrence in teleosts, including cichlids, puffer fish, medaka, ayu, cyprinids, seabreams, and eels [19], [43], [58], [65]–[70]. As shown in Figure 2B, non-duplicated Rh2 genes of freshwater eels clustered with those of moray eels, and together formed a monophyletic group. It is unclear whether freshwater eels failed to undergo Rh2 duplication or subsequently lost one of the duplicated genes; further study in both freshwater and marine eels will be needed to clarify this issue.

The phylogenetic tree of eels and the λ_max_ values of rod cells suggest that different visual abilities may influence the migratory behaviors and habitat choices of eels. As shown in Figure 2D, the rod cells of marine and Asian freshwater eels possess shorter λ_max_ spectra than those of European and American freshwater eels. Asian freshwater eels have similar spawning areas and migratory behaviors to their European and American brethren, but differ in their habitat preferences. The blue-shift in visual spectra may help ensure that the giant mottled eel is better adapted to clear water during upstream migration. In conclusion, different migratory behaviors and habitat preferences may reflect divergent visual signal optimization between eels.

## Conclusion

In this study, we measured and identified the spectral sensitivities and opsin expression patterns of *A. marmorata* at different ontogenetic stages. We report that the giant mottled eel is color-blind and possesses blue-shifted scotopic vision during ontogenetic upstream migration, which may be achieved by alternative chromophore usage and amino acid substitutions located distally to the retinal binding pocket. This unique visual system characteristic, a novel finding among eel species, may influence its upstream migration behaviors and habitat choices. The samples used in this study were collected in Taiwan, yet *A. marmorata* is distributed widely in the Indo-Pacific, from East Africa to French Polynesia, and from southeastern Asia to southern Japan. Is this unique visual system characteristic common to all giant mottled eels, or specific to the population in Taiwan? Future sampling from the Indian Ocean or southeastern Asia will be required to answer this question.

## Supporting Information

Figure S1 **Representative absorbance spectra of rod and cone cells.**
(PDF)

Figure S2 **Protein modeling of known tuning sites in Rh1f and Rh1d.**
(PDF)

Figure S3 **Protein modeling of putative tuning sites in Rh1f and Rh1d.**
(PDF)

Figure S4 **Light spectra (in the air and underwater) of the indicated sampling locations.**
(PDF)

Figure S5 **Rh1 ancestral protein sequences.**
(PDF)

Table S1 **Sample sizes of eels at different stages used for MSP and QPCR measurements.**
(PDF)

Table S2 **Primers used in this study.**
(PDF)

Table S3 **The accession numbers of genes used for the phylogenetic analysis.**
(PDF)

Table S4 **The A1/A2 chromophore ratios of rod cells in eels of different stages.**
(PDF)
