# Supplementary material for: The Giant Mottled Eel, Anguilla marmorata, Uses Blue-Shifted Rod Photoreceptors during Upstream Migration
Source: PLoS One. 2014 Aug 7;9(8):e103953. doi: 10.1371/journal.pone.0103953 (PMC4125165; doi:10.1371/journal.pone.0103953)
Supplement: Figure S1 — Representative absorbance spectra of rod and cone cells. (PDF) [file pone.0103953.s001.pdf]

(A)

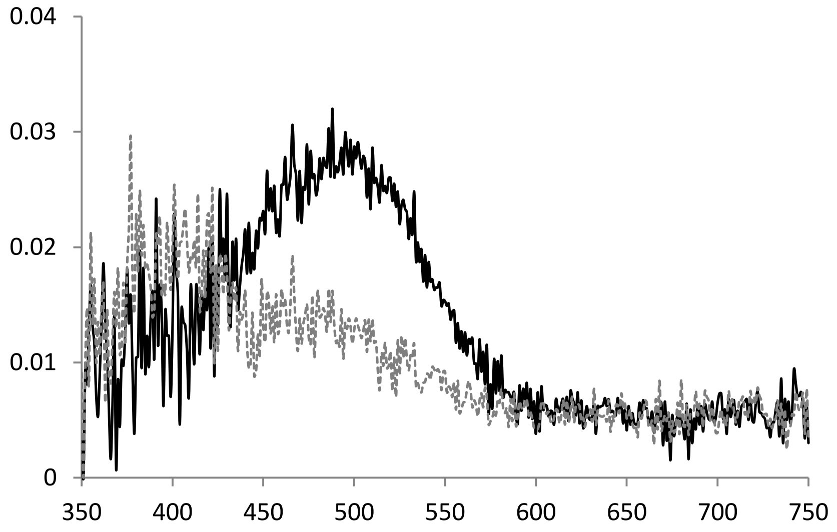

(B)

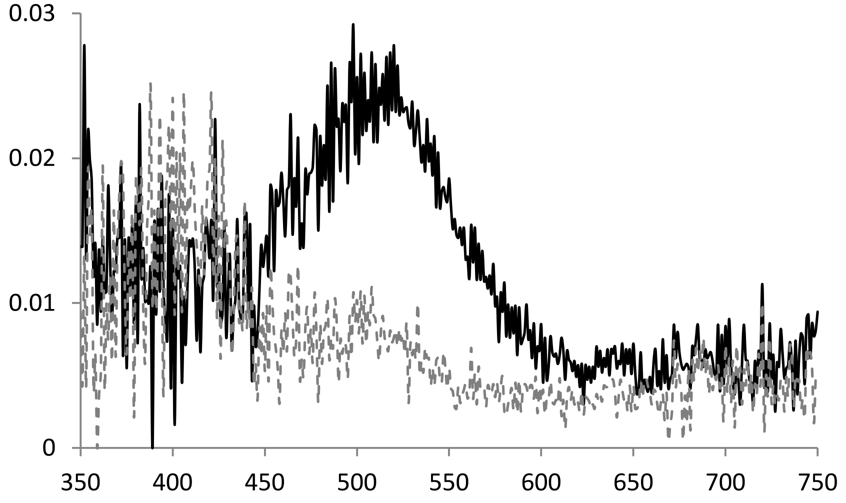

Figure S1 Representative absorbance spectra of rod (A) and cone (B) cells of *A. marmorata*. Black lines: absorbance spectra of photoreceptor cells before bleaching; gray lines: absorbance spectra of photoreceptor cells after bleaching.
