# Supplementary figures and images for: The Giant Mottled Eel, Anguilla marmorata, Uses Blue-Shifted Rod Photoreceptors during Upstream Migration
Source: PLoS One. 2014 Aug 7;9(8):e103953. doi: 10.1371/journal.pone.0103953 (PMC4125165; doi:10.1371/journal.pone.0103953)

D83N

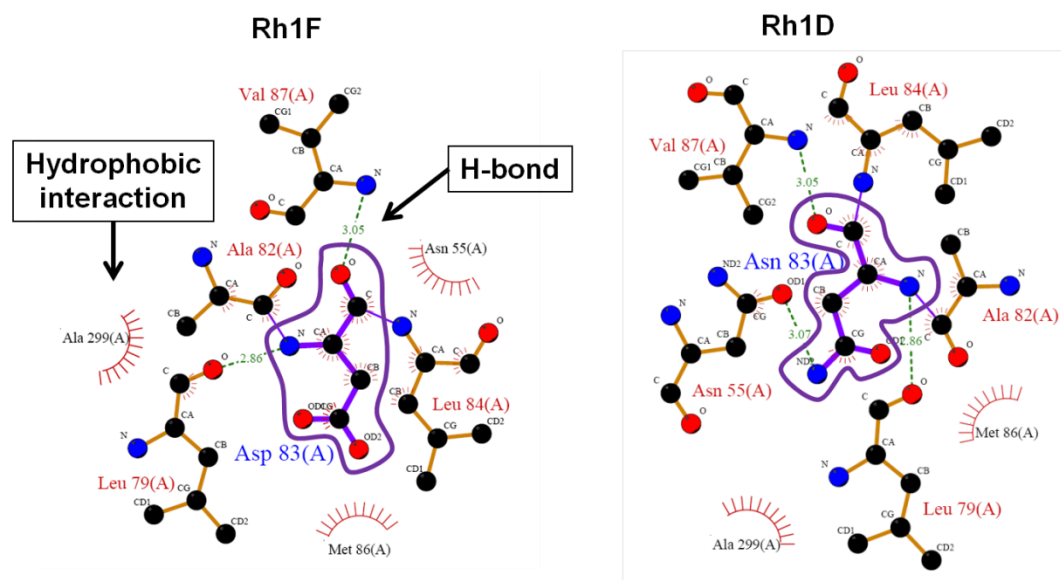

(a) A292S

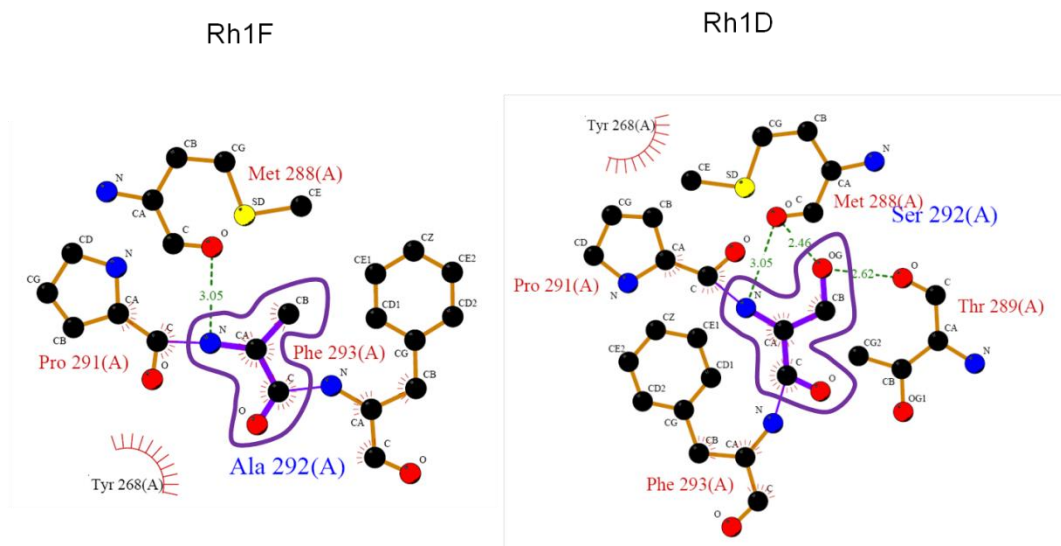

**Figure S2** Protein modeling of (a) D83N and (b) A292S tuning sites in Rh1f and Rh1d.

Supplement: Figure S2 — Protein modeling of known tuning sites in Rh1f and Rh1d. (PDF) [file pone.0103953.s002.pdf]
