# Supplementary material for: The Giant Mottled Eel, Anguilla marmorata, Uses Blue-Shifted Rod Photoreceptors during Upstream Migration
Source: PLoS One. 2014 Aug 7;9(8):e103953. doi: 10.1371/journal.pone.0103953 (PMC4125165; doi:10.1371/journal.pone.0103953)
Supplement: Figure S3 — Protein modeling of putative tuning sites in Rh1f and Rh1d. (PDF) [file pone.0103953.s003.pdf]

(a) A124S

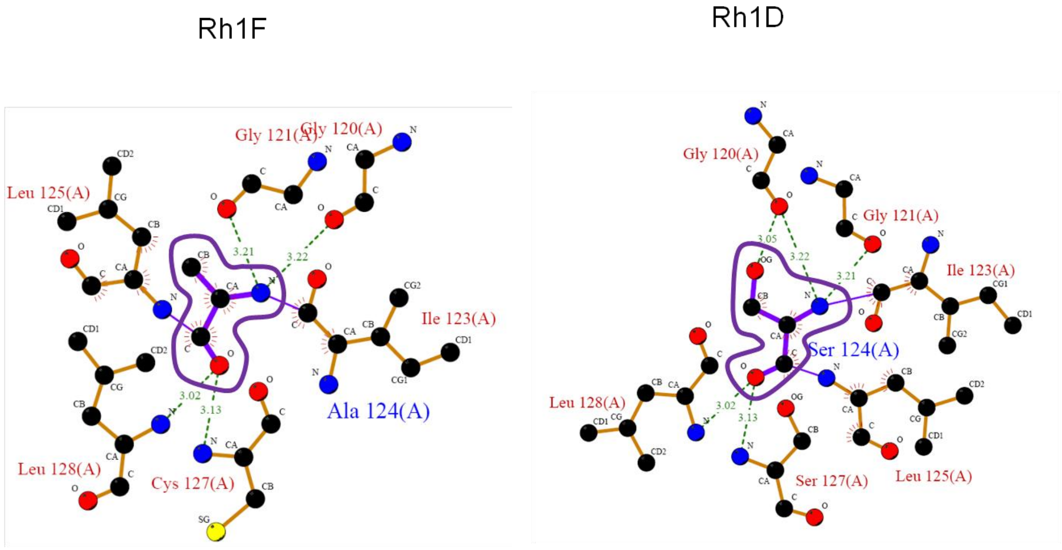

(b) I189V

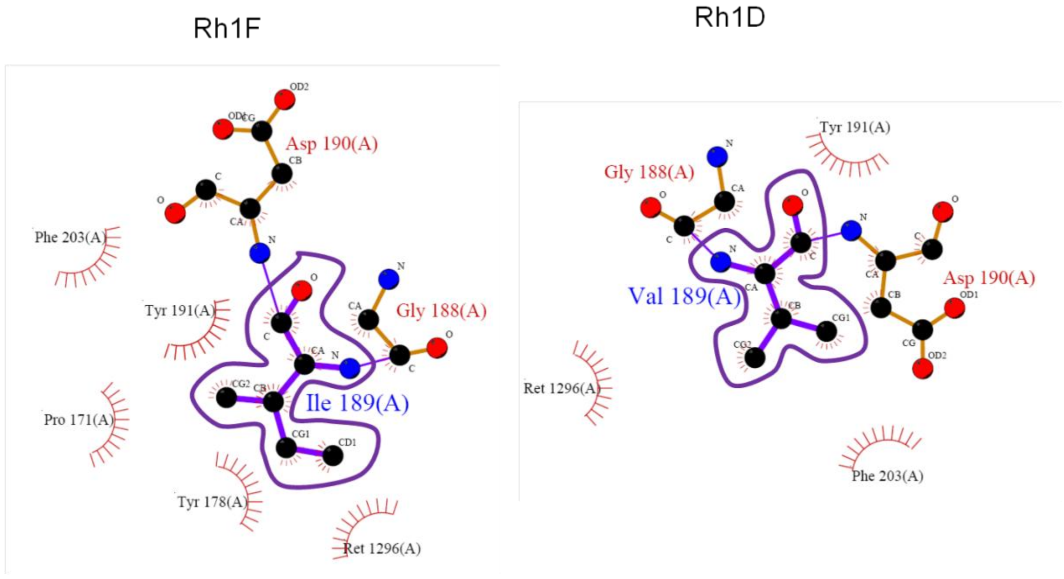

Figure S3 Protein modeling of (a) A124S, (b) I189V, (c) I286V, and (d) L290I tuning sites in Rh1f and Rh1d.

(c)I286V

Rh1F

Rh1D

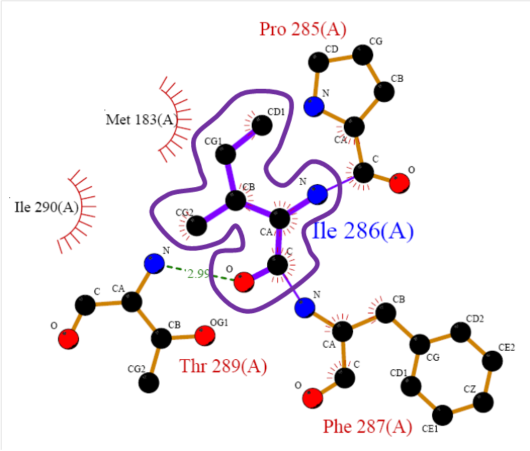

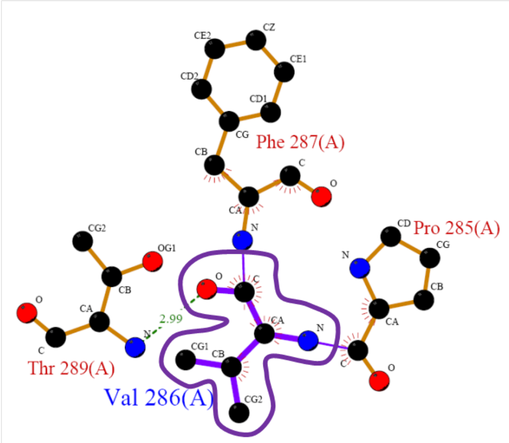

(d)L290I

Rh1F

Rh1D

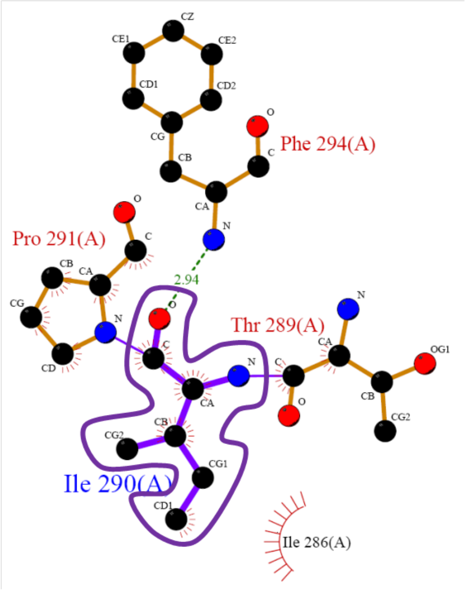

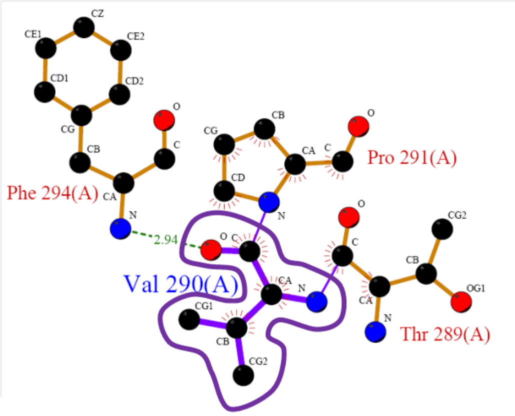

Figure S3 *Continued*.
