# Supplementary material for: The Giant Mottled Eel, Anguilla marmorata, Uses Blue-Shifted Rod Photoreceptors during Upstream Migration
Source: PLoS One. 2014 Aug 7;9(8):e103953. doi: 10.1371/journal.pone.0103953 (PMC4125165; doi:10.1371/journal.pone.0103953)
Supplement: Figure S4 — Light spectra (in the air and underwater) of the indicated sampling locations. (PDF) [file pone.0103953.s004.pdf]

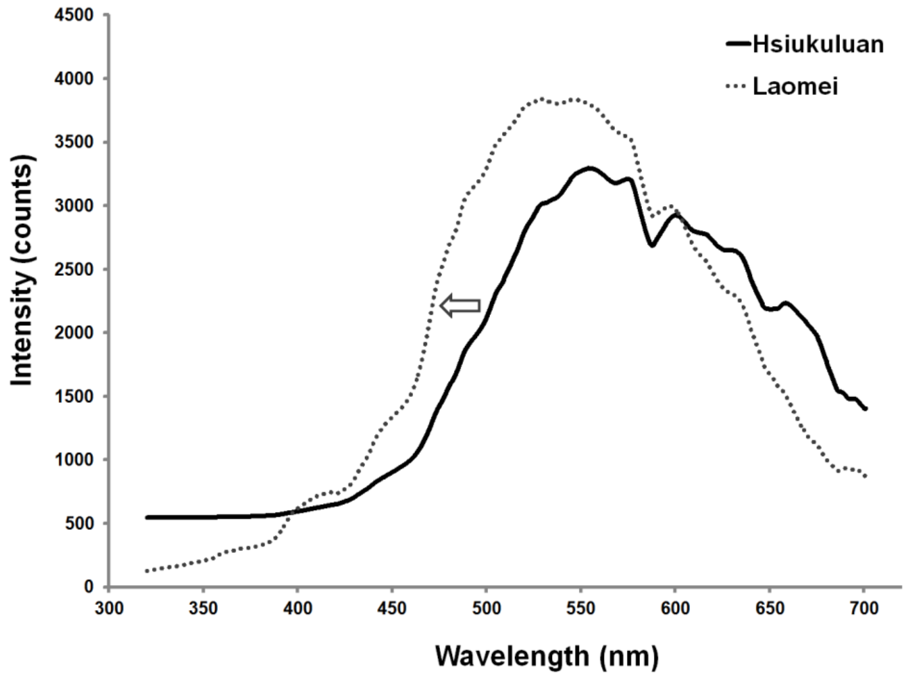

Figure S4 Light spectra of the indicated sampling locations measured underwater at a depth of about 30 cm. The light spectra (wavelength range: 320-700nm) of two sampling localities are shown: the upper basin of the Laomei Stream (dashed line) and the estuary of the Hsiukuluan River (solid line). Notice the obvious blue shift of spectrum (< 550 nm) of Laomei site, as indicated by the open arrow sign pointed to the left of the panel, when comparing with that of Hsiukulan River. Spectra were measured using a USB 2000 spectrometer attached to a PC running SpectraSuite Spectrometer Operating software (Ocean Optics, Dunedin, Florida, USA). The spectrometer was attached to a sensor which is connected to a fiber optic probe with 100 μm aperture. Spectra were obtained by running average with every 30 data points.
