# Supplementary material for: The Giant Mottled Eel, Anguilla marmorata, Uses Blue-Shifted Rod Photoreceptors during Upstream Migration
Source: PLoS One. 2014 Aug 7;9(8):e103953. doi: 10.1371/journal.pone.0103953 (PMC4125165; doi:10.1371/journal.pone.0103953)
Supplement: Figure S5 — Rh1 ancestral protein sequences. (PDF) [file pone.0103953.s005.pdf]

```
Rh1Ancestor
MNGTEGPNFYVPMSNATGVVRSPFEYPQYYLAEPWAYSALAAYMFFLIIA [ 50]
GFPINFLTLYVTIEHKKLRTPLNYILLNLAVADLFMVFGGFTTTMYTSMH [100]
GYFVFGPTGCNLEGFFATLGGEIALWSLVVLAIERWVVVCKPMSNFRFGE [150]
NHAIMGVVFTWIMALACAVPPLFGWSRYIPEGMQCSCGIDYYTRKPEVNN [200]
ESFVIYMFIVHFTIPLTVISFCYGRLVCTVKEAAAQQQESETTQRAEREV [250]
TRMVVIMVISFLVCWVPYASVAWYIFTHQGSNFGPVFMTVPSFFAKSSAI [300]
YNPLIYICMNKQFRNCMITTLCCGKNPFEEEEGASTTASKTEASSVSSSS [350]
VSPA* [355]
```

Figure S5 Rh1 ancestral protein sequences.
