# Supplementary material for: The Giant Mottled Eel, Anguilla marmorata, Uses Blue-Shifted Rod Photoreceptors during Upstream Migration
Source: PLoS One. 2014 Aug 7;9(8):e103953. doi: 10.1371/journal.pone.0103953 (PMC4125165; doi:10.1371/journal.pone.0103953)
Supplement: Table S1 — Sample sizes of eels at different stages used for MSP and QPCR measurements. (PDF) [file pone.0103953.s006.pdf]

Table S1 Sample sizes of eels at different stages used for MSP and QPCR measurements.

	Glass	Cultured yellow	Wild yellow
MSP	3	4[a]	2[b]
QPCR	6	4[a]	2[b]

[a, b] MSP and QPCR were analyzed from the same cultured and wild yellow specimens (one eye was used for MSP and the other for QPCR).
