# Supplementary material for: The Giant Mottled Eel, Anguilla marmorata, Uses Blue-Shifted Rod Photoreceptors during Upstream Migration
Source: PLoS One. 2014 Aug 7;9(8):e103953. doi: 10.1371/journal.pone.0103953 (PMC4125165; doi:10.1371/journal.pone.0103953)
Supplement: Table S2 — Primers used in this study. (PDF) [file pone.0103953.s007.pdf]

Table S2 Primers used in this study.

	PCR primer sequence (5’ to 3’)
Exon 4 of cone opsins	
OPF[a]	5’- GCG AAT TCG CNT CNA CNC ARA ARG ANG A-3’
OPR[a]	5’- GCA AGC TTA CRT ANA TNA YNG GRT TRT A-3’
Rh1	
AjRodP1[b]	5’- ATG AAT GGC ACA GAG GGA CCT AAY TTC T-3’
AjRodP2[b]	5’- GTG TCC TGT GAG ACA AGG TTT ATG C-3’
AjRodP3[b]	5’- TTT GTG GTG GGT CGG TGC CTT ATG C-3’
RACE	
AmBlueEx4F	5’- AGA TGG TGG TGG TGA TGG TGC TGG GCT TC C-3’
AmBlueEx4R	5’- GCG TAC GGC AGC CAG CAG ACC AGG AA-3’
AmG02ex4F	5’- TGG TCA TCT TGA TGG TGC TGG GTT TCC TGA TAG-3’
AmG02ex4R	5’- TGC GAC TCC CTT GTT AAA GAA GAT GTA GGC AGC AT-3’
House-keeping genes	
ARPF	5’-TCG AAG CAC TGC AAA GAT GCC SAG GG-3’
ARPR	5’-GCC AGG ACY CTC TTG TAY CCA TTG ATG-3’
CytbF	5’-CAC AAA TCC TTA CAG GAC TAT TCC TAG-3’
CytbR	5’-GTA AAG GTA TGA GCC GTA GTA AAG-3’
Real-time PCR	
AmQ_Rh1dF	5’- ACT GCA TGA TCA CCA CCT TGT T -3’
AmQ_Rh1dR	5’- TGC TCC CTC TTC CTC CTG AAA -3’
AmQ_Rh1fF	5’- TCA CCC ACC AGG GAA GCA -3’
AmQ_Rh1fR	5’- CAA AGA AGG CTG GAA TGG TCA T -3’
AmQ_Rh2F	5’- ACC TAG GAG AAC CGT GGC AGT A -3’
AmQ_Rh2R	5’- GGG AAG CCA AAG CAG ATG AG -3’
AmQ_S2F	5’- CGT CTG CAC CTC CCA GTA CA -3’
AmQ_S2R	5’- TGC CAC GGC CAG GTT AAC -3’
AmQ_CytBF	5’- CCG CAA CTT ACA TGC AAA CG -3’
AmQ_CytBR	5’- AAG TCC TCG GGC AAT GTG AA -3’
AmQ_APRF	5’- GGG CTC GGT CCT GAG AAG AC -3’
AmQ_APRR	5’- TGG TTC CTC TGG AGA TCT TGG T -3’

a. from (Carleton *et al.* 2000; Hisatomi *et al.* 1994)

b. from (Weltzien *et al.* 2005)
