# Supplementary material for: The Giant Mottled Eel, Anguilla marmorata, Uses Blue-Shifted Rod Photoreceptors during Upstream Migration
Source: PLoS One. 2014 Aug 7;9(8):e103953. doi: 10.1371/journal.pone.0103953 (PMC4125165; doi:10.1371/journal.pone.0103953)
Supplement: Table S3 — The accession numbers of genes used for the phylogenetic analysis. (PDF) [file pone.0103953.s008.pdf]

Table S3 The accession numbers of genes used for the phylogenetic analysis. Fish visual pigments are encoded by the following five classes of opsin genes: rhodopsin (Rh1) in rod cells, and UV-sensitive (short-wavelength sensitive 1; SWS1), blue-sensitive (short-wavelength sensitive 2, SWS2), green-sensitive (rhodopsin-like, Rh2), and red-sensitive (middle/long-wavelength sensitive; M/LWS) in cone cells.

Species	Gene	Accession numbers
Opsin		
Anguilla marmorata (Giant mottled eel)	Rh1d	KJ462781*
	Rh1f	KJ462782*
	Rh2	KJ462783*
	SWS2	KJ462784*
Anguilla anguilla (European eel)	Rh1d	AJ249203
	Rh1f	AJ249202
	Rh2	FJ515778
Anguilla japonica (Japanese eel)	Rh1d	AJ249203
	Rh1f	AJ249202
Conger myriaster (conger eel)	Rh1d	AB043818
	Rh1f	AB043817
Cyprinus carpio (common carp)	SWS2	AB113668
Danio rerio (zebrafish)	Rh2-1	AB087805
	Rh2-1	AB087806
	Rh2-3	AB087807
	Rh2-4	AB087808
	SWS2	NM131192
Geotria australis (pouched lamprey)	SWS2	AY366492
Gymnothorax favagineus (moray eel)	Rh1	HQ444181
	Rh2A	HQ444186
	Rh2B	HQ444187
Latimeria chalumnae (coelacanth)	Rh1	AF131253
	Rh2	AF131258
Dimidiochromis compressiceps (cichlid)	Rh1	AY775059
	SWS2A	AF247113
	SWS2B	AF247117
Melanochromis vermivorus (cichlid)	Rh2Aα	DQ088631
	Rh2Aβ	DQ088634
	Rh2B	DQ088646

Species	Gene	Accession numbers
Opsin		
Metriaclima zebra (cichlid)	Rh1	AY775114
	SWS2A	AF247114
	SWS2B	AF247118
Pseudotropheus acei (cichlid)	Rh2Aα	DQ088630
	Rh2Aβ	DQ088633
	Rh2B	DQ088645
Rhinomuraena quaesita (Ribbon moray)	Rh1	HQ444180
	Rh2A	HQ444184
	Rh2B	HQ444185
Mitochondria		
Anguilla anguilla (European eel)	*cytochrom b*	NC_006531
Anguilla bicolor (Bicolor eel)	*cytochrom b*	NC_006534
Anguilla japonica (Japanese eel)	*cytochrom b*	AF479272
Anguilla marmorata (Giant mottled eel)	*cytochrom b*	NC_006540
Anguilla rostrata (American eel)	*cytochrom b*	NC_006547
Conger myriaster (Conger eel)	*cytochrom b*	NC_002761
Gymnothorax favagineus (moray eel)	*cytochrom b*	EU085374
Rhinomuraena quaesita (moray eel)	*cytochrom b*	AP010844

* This study
