# Supplementary material for: The Giant Mottled Eel, Anguilla marmorata, Uses Blue-Shifted Rod Photoreceptors during Upstream Migration
Source: PLoS One. 2014 Aug 7;9(8):e103953. doi: 10.1371/journal.pone.0103953 (PMC4125165; doi:10.1371/journal.pone.0103953)
Supplement: Table S4 — The A1/A2 chromophore ratios of rod cells in eels of different stages. (PDF) [file pone.0103953.s009.pdf]

Table S4 The A1/A2 chromophore ratio of rod cells in eels of different stages. The ratios were determined from template fitting by microspectrophotometry.

Developmental stage	Rod cell chromophore type		
	A1 type	A2 type	A1/A2 ratio
Glass eel	28	9	3.11
Cultured yellow eel	28	6	4.67
Wild yellow eel[a]	21	10	2.10

[a] Chi-square test was used to demonstrate only a slight significant difference (p value = 0.047) in the A1 and A2 types between wild and cultured yellow eel.
